# Higher Body Iron Is Associated with Greater Depression Symptoms among Young Adult Men but not Women: Observational Data from the Daily Life Study

**DOI:** 10.3390/nu7085270

**Published:** 2015-07-23

**Authors:** Aimee C. Richardson, Anne-Louise M. Heath, Jillian J. Haszard, Maria A. Polak, Lisa A. Houghton, Tamlin S. Conner

**Affiliations:** 1 Department of Psychology, University of Otago, Dunedin 9054, New Zealand; E-Mails: aimee.richardson@otago.ac.nz (A.C.R.); mariapolak@psy.otago.ac.nz (M.A.P.); tconner@psy.otago.ac.nz (T.S.C.); 2 Department of Human Nutrition, University of Otago, Dunedin 9054, New Zealand; E-Mails: jill.haszard@otago.ac.nz (J.J.H.); lisa.houghton@otago.ac.nz (L.A.H.)

**Keywords:** iron, depressive symptoms, daily diary method, micronutrients, mood, young adults

## Abstract

Studies investigating possible associations between iron status and mood or depressive symptoms have reported inconsistent results. However, they have neither used body iron to measure iron status nor measured mood using daily measures. We investigated whether body iron was associated with depressive symptoms, daily mood, daily tiredness, difficulty concentrating, and stress in young adult women and men. Young adult (17–25 years) women (*n* = 562) and men (*n* = 323) completed the Center for Epidemiologic Studies Depression Scale, then reported negative and positive mood, and other states daily for 13 days. Non-fasting venous blood was collected to determine hemoglobin, serum ferritin and soluble transferrin receptor (to calculate body iron), C-reactive protein, and alpha-1-acid glycoprotein concentration. Regression models tested linear associations between body iron and the outcome variables, controlling for possible confounders. No associations were found between body iron and the outcome variables in women. However, higher body iron was associated with more depressive symptoms in men (3.4% more per body iron mg/kg; 95% confidence intervals (CI): 0.8%, 5.9%). In young adult women, body iron is unlikely to be associated with significant deficits in mood or depressive symptoms. However, higher body iron may be associated with more depressive symptoms in young adult men.

## 1. Introduction

Iron deficiency is the most prevalent micronutrient deficiency worldwide [1], yet the psychological consequences of iron deficiency remain unclear. Iron is a co-factor in a number of reactions, including the synthesis of tyrosine, a precursor to the neurotransmitters dopamine and norepinephrine; and tryptophan, a precursor to the neurotransmitter serotonin [2]. These neurotransmitters have been implicated in the traditional monoamine hypothesis of depression which posits that low dopamine, norepinephrine, and serotonin concentrations may result in depression [3]. More recently, the glutamate hypothesis of depression has posited that dysregulation of the glutamatergic system results in depression [4]. Iron is a cofactor for the reaction leading to the production and secretion of glutamate [5]. Globally, depression is the leading cause of disability [6], affecting 9% of adults in the United States [7]. Whether poorer iron status is associated with depression is therefore of particular interest, both scientifically and clinically.

Anemia [8,9] and iron deficiency anemia [10] have both been associated with an increased risk of depression, although the direction of the association is not clear [11] and may be confounded by disease [12]. Low iron status in the absence of anemia is considerably more common than iron deficiency anemia in the USA [13] and New Zealand (NZ) [14]; however it is even less clear whether low iron status in the absence of anemia is associated with lower mood. Some studies have reported an association between low iron status and decreased well-being [15] or increased depressive symptoms [12,16,17] in healthy adults. However, only one of these studies [16] used a measure of inflammation to address serum ferritin concentrations likely to be elevated by infection, and two of the studies used non-standard methods for determining iron status [15] and depressive symptoms [17]. A number of other studies have reported no association between iron status and well-being or depressive symptomatology [18,19,20,21,22,23].

To date, very few studies have investigated whether iron status may have mood effects in men [12,18,24], and none have used mood data collected in “near to real time” rather than retrospectively. Mood measured using real-time data capture techniques is less susceptible to memory biases than mood measured using retrospective measures, and is potentially more sensitive for detecting mood differences [25,26]. Moreover, no studies have rigorously accounted for the effects of inflammation on serum ferritin concentration, or used body iron to determine iron status. Body iron enables a wide range of iron status to be described in a single continuous variable from severe deficiency to repletion [27].

The primary aim of this study was to determine whether body iron was associated with depressive symptoms or daily mood in a large non-clinical sample of young adults. Secondary aims were to determine whether body iron was associated with other self-reported daily states, specifically tiredness, difficulty concentrating, or stress; and whether the different stages of iron deficiency (low iron stores, non-anemic iron deficiency, iron deficiency anemia) were associated with depressive symptoms, daily mood, or other daily states (tiredness, difficulty concentrating, or stress).

## 2. Experimental Section

### 2.1. Study Design and Procedure

Participants were from Dunedin, New Zealand, and recruited as part of the Daily Life Study, a large cross-sectional study of well-being in young adults (for previous publications from this data set see references [28,29]). Each participant participated in the study for a two week period. The data were collected in 2011, 2012, and 2013. At baseline, participants completed the Center for Epidemiologic Studies Depression Scale (CES-D; [30]) and provided demographic information (age, gender, ethnicity). For the following thirteen days, they completed an Internet-based daily diary measuring self-reported positive and negative mood, tiredness, difficulty concentrating, stress, alcohol consumption, and physical activity. On day 14, a non-fasting venous blood sample was drawn to determine iron status, height and weight were measured using standardized procedures [31], and a final questionnaire was administered to collect information on use of oral-contraceptive and anti-depressant or mood stabilizing medication. The study was approved by the University of Otago Human Ethics Committee (#10/177) and was registered with the Australian New Zealand Clinical Trials Registry (ACTRN12613000773730).

### 2.2. Participants

Participants were students at the University of Otago. Although they were recruited predominantly through the Department of Psychology experimental participation scheme (and reimbursed with partial course credit based on a worksheet exercise); efforts were made to recruit a wide range of students by using flyers, an on-campus employment agency, and Department of Human Nutrition classes (in which case participants were reimbursed with a small payment). Inclusion criteria were: 17–25 years of age, enrolled at least part-time at the University of Otago, access to a mobile phone and the Internet. Exclusion criteria were: completed fewer than 7 daily diaries, did not provide a complete blood sample, did not complete the final questionnaire, presence of inflammation (C-Reactive Protein (CRP) ≥ 10 mg/L [32], or alpha-1-acid glycoprotein (AGP) > 1 g/L [33]), and currently taking antidepressant or mood stabilizing medication. No formal sample size calculation was carried out, but recruitment of participants in early 2011, 2012, and 2013 enabled identification of at least 10 female participants with iron deficiency anaemia (the smallest category of iron deficiency)—at least 10 cases are recommended for each predictor in a regression model [34], so examination of associations with iron status in women was possible in this sample.

### 2.3. Psychological Measures

*Depressive symptomatology* was measured at baseline using the Center for Epidemiologic Studies of Depression Scale (CES-D [30]), a 20-item validated measure of depressive symptoms in the past week designed for use in the general population. Missing data points (*n* = 6 of 17,700, 0.03%) were substituted with the participant’s mean response for the items answered [35]. Reports were summed for a continuous measure of depressive symptoms ranging from 0 to 60. For descriptive purposes, a total score of 16 or more (out of a possible 60) was used to indicate presence of high depressive symptomatology [30].

Daily mood was measured in the daily diary using an 18-item measure assessing positive mood and negative mood. The positive and negative mood scales each contained three subscales of low, medium and high activation (*i.e.*, arousal) states to capture the full range of possible moods [36]. There were nine items assessing positive mood (low activation: *content*, *calm*, *relaxed*; medium activation: *pleasant*, *happy*, *cheerful*; high activation: *excited*, *energetic*, and *enthusiastic*) and nine items assessing negative mood (low activation: *dejected*, *sad*, *unhappy*; medium activation: *nervous*, *tense*, *anxious*; high activation: *irritable*, *hostile*, and *angry*). Participants answered each item based on how they felt “today” on a 5-point scale of 0 (not at all) to 4 (extremely). Mood items were presented in the same randomized order each day. This model has been previously validated [36]. Reliability analyses showed that there was sufficient reliability to warrant combining the nine positive adjectives into a scale (α = 0.82) and sufficient reliability for combining the nine negative adjectives into a scale (α = 0.78; alpha reliabilities for nested data computed using recommended guidelines from Nezlek [37]). Daily positive mood and negative mood were averaged across the diaries. In our sample, the positive (*r* = −0.39, *p* < 0.001) and negative mood scales (*r* = 0.46, *p* < 0.001) were both significantly correlated with the CES-D.

Additional states—daily tiredness, difficulty concentrating, and stress—were also measured in the daily diaries. Two unstandardized questions asked participants to rate the extent to which they “felt tired” and “had difficulty concentrating” that day using 5-point scales from 0 (not at all) to 4 (very). Participants rated how much stress they had been under that day on a 5-point scale from 0 (no stress) to 4 (a great deal of stress). Reports were averaged across the diaries.

### 2.4. Biochemical Assessment

Venous blood was drawn on the final day of the study using a 2 mL ethylenediaminetetraacetic acid (EDTA) vacutainer (Becton Dickinson, Franklin-Lakes, NJ, USA) to determine hemoglobin, and a 10 mL additive-free vacutainer (Becton Dickinson, Franklin Lakes, NJ, USA) to determine serum ferritin, soluble transferrin receptor, C-reactive protein (CRP), and alpha-1-acid glycoprotein (AGP). Samples used for analysis of hemoglobin were transported to Southern Community Laboratories (Dunedin, NZ) for analysis. Samples for serum analysis were refrigerated and then separated within 4 h of collection at the Department of Human Nutrition Trace Element Laboratory (University of Otago, Dunedin, NZ. Aliquots of serum were stored in cryovials at −80 °C until analysis.

Hemoglobin (Hb) was analyzed using a Sysmex XE-5000 automated hematology analyzer (Roche Diagnostics, Indianapolis, IN, USA) (Coefficient of Variation, CV = 0.8%). Serum ferritin (SF) was measured using an immunoturbidimetric assay with a Roche Hitachi Cobas C311 automated analyzer (Roche Diagnostic, Mannheim, Germany). Quality-control sera at 119 µg/L and 223 µg ferritin/L were assayed. The analyzed means (standard deviation, SD; CV) were 123.0 µg ferritin/L (2.5 µg/L; 2.0%) and 231.5 µg ferritin/L (10.0 µg/L; 4.3%). Control pooled-serum samples were analyzed during each batch of analyses to assess inter-batch variability (CV = 0.9%). Soluble transferrin receptor (sTfR) was assessed using an enzyme immunoassay with a Roche Hitachi Cobas C311 automated analyzer (Roche Diagnostic, Mannheim, Germany). Quality-control sera at 2.3 mg/L and 7.2 mg/L were assayed. The analyzed means (SD; CV) were 2.4 mg/L (0.1 mg/L; 4.0%) and 7.3 mg/L (0.7 mg/L; 1.0%). Control pooled-serum samples were analyzed during each batch of analyses to assess inter-batch variability (CV = 3.1%). Soluble transferrin receptor concentrations were later converted to be equivalent with the Flowers assay using the following equation: Flowers sTfR = 1.5 × Roche sTfR + 0.35 mg/L [13]. Serum C-reactive protein (CRP) was measured using an immunoturbidimetric assay with a Roche Cobas C502 analyzer (Roche Diagnostics, Mannheim, Germany). Quality-control sera at 11.8 mg/L were assayed. The analyzed mean (SD; CV) was 11.5 mg/L (0.4 mg/L; 3.5%). Control pooled-serum samples were analyzed during each batch of analyses to assess inter-batch variability (CV = 4.6%). A cut-off of ≥10 mg/L was used to indicate the presence of inflammation [32]. Alpha-1-acid glycoprotein (AGP) was measured using an immunoturbidimetric assay with a Roche Hitachi Cobas C311 automated analyzer (Roche Diagnostic, Mannheim, Germany). Quality-control sera at 0.7 g/L and 1.3 g/L were assayed. The analyzed means (SD; CV) were 0.7 g/L (0.02 g/L; 2.4%) and 1.3 g/L (0.03 g/L; 2.5%). Control pooled-serum samples were analyzed during each batch of analyses to assess inter-batch variability (CV = 1.2%). A cut-off of ≥1 g/L was used to indicate the presence of inflammation [33].

Body iron was calculated using the following formula [13]:

$$\left[\text{Body iron }\left(\frac{\text{mg}}{\text{kg}}\right)=-\;\frac{\;\left[\text{log}10\left(\text{sTfR x}\frac{1000}{\text{ferritin}}\right)-2.8229\right]}{0.1207}\right]$$

Stages of iron deficiency were defined as iron sufficient, low iron stores, non-anemic iron deficiency, or iron deficiency anemia (see Table 1).

**Table 1:** Criteria used to indicate the stage of iron deficiency of participants.

Stage of Iron Deficiency	Cut-off Values for Females Aged 17 years and above	Cut-off Values for Males Aged 19 Years and above ^1^
Iron sufficient	SF ≥ 20 µg/L ^2^ and body iron ≥ 0 mg/kg ^3^	SF ≥ 20 µg/L ^2^ and body iron ≥ 0 mg/kg ^3^
Low iron stores	SF < 20 µg/L ^2^ and body iron ≥ 0 mg/kg ^3^	SF < 20 µg/L ^2^ and body iron ≥ 0 mg/kg ^3^
Non-anemic iron deficiency	Body iron < 0 mg/kg ^3^ and Hb ≥ 120 g/L ^4^	Body iron < 0 mg/kg ^3^ and Hb ≥ 137 g/L ^4^
Iron-deficiency anemia	Body iron < 0 mg/kg ^3^ and Hb < 120 g/L ^4^	Body iron < 0 mg/kg ^3^ and Hb < 137 g/L ^4^

SF = serum ferritin; Hb = hemoglobin; ^1^ For males aged 18 years or under a Hb cut-off of <136 g/L was used; ^2^ Cut-off value obtained from reference [38]; ^3^ Cut-off value obtained from reference [39]; ^4^ Cut-off value obtained from reference [40].

### 2.5. Sociodemographic and Health Variables

Data on age, ethnicity, and gender were collected by self-report questionnaire at baseline. Ethnic categories were based on the most populous Level-1 New Zealand census categories (European, Māori, Pacifika, Asian, Indian, Middle Eastern/Latin American/African, Other), which we descriptively grouped into European, Māori/Pacifika, Asian, and Other. Body mass index (BMI; kg/m^2^) was calculated using height and weight measures obtained on the final day of the study using standardized procedures [31]. With the participant wearing no shoes, height was measured using calibrated equipment (Seca 216 stadiometer, Seca North America, Chino, CA, USA), and weight using a bioimpedance scale (TBF-310, Tanita Corporation, Arlington Heights, IL, USA). Average weekly alcohol consumption was calculated using data from the daily diaries. In each daily diary, participants were asked to report the number of standard drinks consumed in the previous 24 h (1 standard drink = 10 g of ethanol: 330 mL can or bottle of normal strength beer or cider (4% alcohol); 100 mL glass of wine (12.5% alcohol); or 30 mL of liquor (40% alcohol; straight or in a mixed drink)). Mean daily physical activity was calculated using data from the daily diaries. Participants were asked to report the total minutes of vigorous or moderate physical activity (“activities that take hard or moderate physical effort and make you breathe much harder or somewhat harder than normal” [41]) completed in the previous 24 h. To control for varying stress levels during the semester, the month in the semester when the participant started the study was entered as a covariate. For all participants, current prescription of anti-depressant or mood stabilizing medications and, for women, current use of oral contraceptive agents (OCA), were identified in the final questionnaire.

### 2.6. Statistical Analyses

All analyses were run using SPSS (Version 22.0; IBM Corp. Released 2010. IBM SPSS Statistics for Windows, Armonk, NY, USA.). A *p*-value < 0.05 was considered to indicate statistical significance. Before conducting any analyses, histograms were created for the outcome variables (depressive symptoms, negative mood, positive mood, tiredness, difficulty concentrating, and stress), and log transformations were used where the variable was right skewed (a constant of one was added to four zero values). 

A series of multiple regressions were run separately for women and men. This approach allowed us to compute separate effect sizes for women and men, and also to control for OCA use in women. Controlling for OCA use is important as previous research has found associations between mood and iron status varied between OCA users and non-users [42]. The first models used body iron as the continuous predictor variable and depressive symptoms (CES-D), negative daily mood, or positive daily mood as continuous outcomes. These were adjusted for BMI (kg/m^2^), weekly alcohol consumption (number of standard drinks/day), physical activity (minutes of moderate or vigorous physical activity/day), CRP (mg/L), AGP (g/L), time of semester (month), and for women, current use of OCA (yes = 1 or no = 0). Subsequent models were repeated with the secondary outcomes: tiredness, difficulty concentrating, and stress. *Post hoc* analyses tested the relation between body iron and the low, medium, and high activation negative mood state subscales, and low, medium, and high activation positive mood state subscales using the same process. This was done because previous studies have linked iron levels to specific mood states, such as medium activation negative mood (anxiety) [43] and high activation positive mood, such as vitality [22]. By testing high, medium, and low activation moods separately, we were able to test these relationships.

A second set of multiple regression models were run with stage of iron deficiency as a categorical predictor of depressive symptoms (and the other outcomes), controlling for the same covariates. Three dummy codes were entered as simultaneous predictors with iron sufficient participants as the reference group. Due to the low-prevalence of iron deficiency among men in our sample, these analyses were run only for women.

Regression coefficients and 95% confidence intervals were calculated and, for ease of interpretation, these were back-transformed and presented as a percentage difference for the log-transformed variables. Residuals were checked for homogeneity of variance and normality.

## 3. Results

There were 1061 participants who met the inclusion criteria. Of these, 176 participants were excluded from the analysis (completed fewer than 7 daily dairies (*n* = 30), did not complete the final questionnaire (*n* = 4), did not provide a complete blood sample (*n* = 19), taking antidepressant or mood stabilizing medication (*n* = 34), elevated inflammatory marker (CRP ≥ 10 mg/L, *n* = 31; AGP > 1 g/L, *n* = 58), leaving a final sample of 885 participants (562 female, 323 men) who completed on average 11.7 of 13 diaries (90% compliance). The participants who were excluded from the study were slightly but significantly older than those who were included in the analyses (mean = 20.6 years compared to 19.7 years; *p* = 0.013). There were no significant differences in gender or ethnicity. 

Female participants had a mean age of 19.4 years, and a mean BMI of 23.5 kg/m^2^ (Table 2). The majority were iron sufficient (79.2%) with only 2.1% of the women in this sample classified as having iron deficiency anemia. Body iron values ranged from −10.7 mg/kg to 13.5 mg/kg, and serum ferritin values ranged from 0.5 µg/L to 229.5 µg/L. The geometric mean of CES-D depressive symptoms score for females was 12.0 (range = 0.0 to 50.0), with 37.3% of the female participants reporting high depressive symptomatology (*i.e.*, ≥16 points on the CES-D). 

Male participants had a mean age of 20.1 years, and a mean BMI of 24.0 kg/m^2^ (Table 2). The majority were iron sufficient (97.5%) with only 0.6% of the men in this sample classified as having iron deficiency anemia. Body iron values ranged from −3.9 mg/kg to 17.7 mg/kg, and serum ferritin values ranged from 5.3 µg/L to 465.2 µg/L. The geometric mean CES-D depressive symptoms score for males was 10.6 (range = 0.0 to 40.0), with 28.8% of the male participants reporting high depressive symptomatology (*i.e.*, ≥16).

After adjusting for covariates (BMI, vigorous or moderate physical activity, weekly alcohol consumption, OCA use, time of semester, CRP, and AGP), body iron did not significantly predict depressive symptoms, negative mood, positive mood, or any of the other outcome variables (tiredness, problems concentrating, and stress) in women (Table 3). The largest of these non-significant effect sizes (for depressive symptoms: 0.2% per mg/kg of body iron) had a confidence interval of −1.3% to 1.7%, suggesting that, at most, a 1 mg/kg higher body iron could be associated with between 1.3% lower depressive symptoms and 1.7% higher depressive symptoms in the female population as a whole. In contrast, men showed a much clearer pattern. After adjusting for the same covariates (except OCA use), body iron significantly predicted depressive symptoms in men, with higher body iron associated with 3.4% (95% CI: 0.8%, 5.9%; *p* = 0.009) higher levels of depressive symptomatology per mg/kg of body iron (Table 3). For men, with one standard deviation higher body iron (*i.e.*, 2.8 mg/kg higher body iron) this effect size would be equivalent to a score on the CES-D scale of 11.7 points compared to the average score of 10.6. Overall, this regression model explained 3.4% of the variance in depressive symptoms. Although body iron was also found to significantly and positively predict stress among men (*p* = 0.041), the relationship between body iron and stress was no longer significant when controlling for depressive symptoms (the regression coefficient, B = 0.018; 95% CI = −0.006, 0.041; *p* = 0.145). Body iron did not significantly predict negative mood, positive mood, tiredness, or problems concentrating in men. 

**Table 2:** Characteristics of female (*n* = 562) and male participants (*n* = 323). ^1^

	**Female Participants**		**Male Participants**	
	Mean ^2^ ± SD	*n* (%)	Mean^2^ ± SD	*n* (%)
Age (years)	19.4 ± 1.43	-	20.1 ± 1.78	-
Ethnicity				
*European*	-	454 (80.8)	-	253 (78.3)
*Māori/Pasifika*	-	16 (2.8)	-	13 (4.0)
*Asian*	-	56 (10.0)	-	35 (10.8)
*Other*	-	36 (6.4)	-	22 (6.9)
Body mass indext (BMI) (kg/m^2^)	23.5 ± 4.14	-	24.0 ± 3.6	-
Alcohol intake (standard drinks/wk) ^3^	3.4 (3.0, 3.8)	-	6.2 (5.3, 7.3)	-
Physical activity (minutes/day) ^3^	21.7 (19.9, 23.6)	-	23.4 (20.4, 26.7)	-
Taking oral contraceptive agent	-	267 (47.5)	-	-
Hemoglobin (g/L)	133.6 ± 8.7	-	154.4 ± 8.9	-
Serum ferritin (µg/L) ^3^	35.6 (33.4, 37.9)	-	94.4 (88.0, 101.4)	-
Soluble transferrin receptor (mg/L)	5.6 ± 1.9	-	5.2 ± 1.3	-
C-reactive protein (CRP) (mg/L)	1.6 ± 1.7	-	1.0 ± 1.3	-
Alpha-1-acid glycoprotein (AGP) (g/L)	0.6 ± 0.2	-	0.7 ± 0.2	-
Body iron (mg/kg)	5.4 ± 3.4	-	9.1 ± 2.8	-
Iron deficiency anemia	-	12 (2.1)	-	2 (0.6)
Non-anemic iron deficiency	-	33 (5.9)	-	1 (0.3)
Low iron stores	-	72 (12.8)	-	5 (1.5)
Iron sufficient	-	445 (79.2)	-	315 (97.5)
Depressive symptoms ^3^ (CES-D ^4^)	12.0 (11.3, 12.7)	-	10.6 (9.9, 11.4)	-
Negative mood ^3,5^	0.6 (0.6, 0.7)	-	0.6 (0.6, 0.7)	-
Positive mood ^5^	2.0 ± 0.5	-	2.1 ± 0.5	-
Tiredness ^5^	1.8 ± 0.7	-	1.5 ± 0.7	-
Problems concentrating ^5^	1.5 ± 0.7	-	1.3 ± 0.7	-
Stress ^5^	1.4 ± 0.60	-	1.3 ± 0.6	-

^1^ Following exclusion of participants who did not meet study criteria: < 17 or > 25 years; did not complete ≥7 daily diaries; no blood sample; unable to analyze blood sample; did not complete final questionnaire; CRP ≥ 10 mg/L or AGP > 1 g/L; taking antidepressant or mood stabilizing medication; ^2^ Data are arithmetic mean ± standard deviation (SD) unless stated otherwise; ^3^ Skewed data are geometric mean (95% confidence interval); ^4^ Centre for Epidemiologic Studies Depression Scale (a score of 16 out of a possible 60 is considered to indicate presence of high depressive symptomatology) [30]; ^5^ On a 5-point scale from 0 to 4.

To better understand the association between body iron and depressive symptoms in men, two further analyses were undertaken. First, CES-D was dichotomized (scores ≥ 16 = at risk of depression, scores < 16 = not at risk of depression [30]). A logistic regression was then run to determine the odds of being depressed for each unit increase of body iron, adjusted for the covariates that were adjusted for in the multiple regression models (Table 3). Body iron was not associated with an increased risk of depression in young men (OR = 1.08, 95% CI 0.98–1.18, *p* = 0.118). Second, body iron was categorized by quintile and an analysis of covariance (ANCOVA) was carried out to determine whether the geometric mean CES-D score differed between different body iron quintiles. This model was adjusted for the covariates as before and an *F*-test was used to see if there were any differences overall, followed by pairwise comparisons when the *F*-test suggested that there were differences. These results are presented in Table 4.

**Table 3:** Association between body iron (mg/kg) and depressive symptoms, mood, and additional outcome variables in women (*n* = 562) and men (*n* = 323). ^1^

	Difference in score for 1 mg/kg increase in body iron ^2^	% Difference in score for 1 mg/kg increase in body iron ^3^	95% CI	*p-*Value
**Women**				
Depressive symptoms ^3^	-	0.200	−1.291, 1.715	0.800
Negative mood ^3^	-	−0.009	−0.598, 0.602	0.979
Positive mood	0.002	-	−0.010, 0.013	0.788
Tiredness	0.0004	-	−0.020, 0.014	0.715
Problems concentrating	−0.002	-	−0.015, 0.016	0.962
Stress	−0.001	-	−0.014, 0.016	0.890
**Men**				
Depressive symptoms ^3^	-	3.355	0.803, 5.866	0.009 **
Negative mood ^3^	-	0.200	−0.896, 1.308	0.665
Positive mood	−0.012	-	−0.032, 0.008	0.244
Tiredness	0.011	-	−0.018, 0.040	0.451
Problems concentrating	0.017	-	−0.010, 0.043	0.220
Stress	0.025	-	0.001, 0.049	0.041 *

^1^ Adjusted for body mass index (BMI), vigorous or moderate physical activity, weekly alcohol consumption, oral contraceptive agents (OCA) use, time of semester, C-reactive protein (CRP), and alpha-1-acid glycoprotein (AGP); ^2^ Normally distributed data are reported as difference in score for 1 unit increase in body iron; ^3^ Skewed data are log−transformed and reported as percentage difference in score for 1 unit increase in body iron; * Significant at *p* < 0.05; ** Significant at *p* < 0.01; 95% CI: 95% confidence intervals.

**Table 4:** Differences in geometric mean depressive symptoms in men (*n* = 323) across body iron quintiles (mg/kg). ^1^

Body Iron Quintiles (mg/kg)	*n*	Geometric Mean Depressive Symptoms	95% CI
I (<7.17)	64	10.0	8.452, 11.79
II (7.17–8.76)	65	10.2	8.591, 12.17
III (8.77–9.94)	64	8.6	7.302, 10.24
IV (9.95–11.17)	65	11.0	9.231, 13.10
V (>11.17)	64	12.8	11.09, 14.85

^1^ Adjusted for body mass index (BMI), vigorous or moderate physical activity, weekly alcohol consumption, time of semester, C-reactive protein (CRP), and alpha-1-acid glycoprotein (AGP); 95% CI: 95% confidence intervals.

**Table 5:** Association between stage of iron deficiency and depressive symptoms and negative mood (compared to iron sufficiency) in women (*n* = 562). ^1^

	Low Iron Stores (*n* = 72)			Non-Anemic Iron Deficiency (*n* = 33)			Iron Deficiency Anemia (*n* = 12)		
	Percent difference in score ^2^	95% CI	*p*-Value	Percent difference in score ^2^	95% CI	*p*-Value	Percent difference in score ^2^	95% CI	*p*-Value
Depressive symptoms	8.872	−6.667, 27.12	0.276	2.122	−18.12, 27.25	0.853	1.613	−28.75, 44.91	0.930
Negative mood ^3^	7.466	0.803, 14.57	0.027*	2.224	−6.667, 12.08	0.633	2.327	−11.66. 18.53	0.759

^1^ Adjusted for body mass indext (BMI), vigorous or moderate physical activity, weekly alcohol consumption, oral contraceptive agents (OCA) use, time of semester, C-reactive protein (CRP), alpha-1-acid glycoprotein (AGP); ^2^ The data are skewed so are log-transformed and reported as percentage difference compared to the iron sufficient category; ^3^ Negative mood items were: dejected, sad, unhappy, nervous, tense, anxious, irritable, hostile, angry; * Significant at *p* < 0.05; 95% CI: 95% confidence intervals.

**Table 6:** Association between stage of iron deficiency and positive mood, and additional variables (compared to iron sufficiency) in women (*n* = 562). ^1^

	Low Iron Stores (*n* = 72)			Non-Anemic Iron Deficiency (*n* = 33)			Iron Deficiency Anemia (*n* = 12)		
	Difference in score ^2^	95% CI	*p*-Value	Difference in score ^2^	95% CI	*p*-Value	Difference in score ^2^	95% CI	*p*-Value
Positive mood ^3^	−0.038	−0.155, 0.078	0.518	−0.057	−0.224, 0.110	0.504	−0.136	−0.405, 0.133	0.321
Tiredness	0.047	−0.125, 0.219	0.591	0.087	−0.160, 0.333	0.489	0.062	−0.335, 0.460	0.758
Problems concentrating	−0.022	−0.183, 0.138	0.786	0.168	−0.061, 0.398	0.150	0.144	−0.226, 0.514	0.446
Stress	0.017	−0.131, 0.165	0.819	−0.076	−0.288, 0.135	0.479	0.073	−0.267, 0.414	0.672

^1^ Adjusted for body mass index (BMI), vigorous or moderate physical activity, weekly alcohol consumption, oral contraceptive agents (OCA) use, time of semester, C-reaction protein (CRP), alpha-1-acid glycoprotein (AGP); ^2^ The data are normally distributed so are reported as difference compared to the iron sufficient category; ^3^ Positive mood items were: content, calm, relaxed, pleasant, happy, cheerful, excited, energetic, enthusiastic; * Significant at *p* < 0.05; 95% CI: 95% confidence intervals.

After adjusting for covariates (as listed above) the two most severe *stages of iron deficiency*, iron deficiency anemia (*n* = 12) and non-anemic iron deficiency (*n* = 33), were not associated with any of the outcome variables in women (Table 5 and Table 6). Participants with low iron stores (*n* = 72) were found to have 7.5% higher negative mood scores than those participants who were iron sufficient (*p* = 0.027), suggesting that women with low iron stores experienced more negative mood. However, because the mean negative mood scores were so low in this sample overall (mean = 0.6), this equated to a difference in negative mood scores of 0.11 points on a 5-point scale between those with low iron stores and those who were iron sufficient (for women not taking OCAs, assuming participation at the start of the semester and a mean value for the continuous covariates). Therefore, this association, while statistically significant, is not practically significant. When the individual negative mood subscales were investigated in a *post hoc* analysis, the association seemed to be largely carried by significantly greater low activation negative mood states (dejected, sad, unhappy) for the participants with low iron stores (B = 7.778%, *p* = 0.037), although high activation mood states (irritable, hostile, angry) were close to statistical significance (B = 6.716%, *p* = 0.054). These effect sizes were of a similar magnitude to the effect size for the negative mood scale overall. There were no other significant associations for those female participants with low iron stores. No model for stages of iron deficiency was conducted for men due to the low prevalence of iron deficiency in this sample.

## 4. Discussion

In this large and comprehensive study, there were no associations between body iron, iron deficiency anemia, or non-anemic iron deficiency and depressive symptomatology or mood in women. Although women with low iron stores reported slightly more negative mood (particularly more sadness) than women who were iron-sufficient, the size of this association was too small to be of practical significance. Surprisingly, higher body iron was found to be associated with higher depressive symptoms among young adult males, although not with higher risk of depression (defined as a CES-D score ≥ 16).

Previous research has reported associations between low iron status and more depressive symptoms and lower well-being among women [12,15,16,17]. However, all but one of these studies were limited by failing to account for inflammation when using serum ferritin to measure iron status (an essential requirement because serum ferritin is an acute phase reactant and is therefore elevated in the presence of inflammation) [12,15,17], and by using only an unstandardized measure of iron status [15] or mood [17]. In contrast, the current study addressed inflammation by excluding participants with elevated concentrations of CRP or AGP, and adjusting for both inflammatory markers in the analyses. We also used comprehensive measures of iron status—both body iron as a continuous measure of iron status, and body iron in combination with Hb concentration to differentiate between non-anemic and anemic iron deficiency; and comprehensive measures of mood—using both retrospective and “near to real time” measures. The null findings for body iron in this large study (*n* = 562 women), alongside previous studies that have reported no association between iron status and depressive symptoms, mood, or well-being [18,19,20,21,22,23], suggest that it is unlikely that depression or clinically important deficits in mood are associated with iron status in apparently healthy young women.

Although women with low iron stores in this study reported more negative mood (particularly sadness) than women who were iron-sufficient, the association was too small to be of practical significance (just 0.1 points on a 5-point scale), and was not found in women with more severe iron deficiency. This is unexpected because the incremental nature of iron deficiency means that participants with non-anemic or anemic iron deficiency would be expected to have even lower, or absent, iron stores, and therefore to show at least the same deficit in mood as those with low iron stores. It is possible that no associations were found with iron deficiency anemia (*n* = 12) and non-anemic iron deficiency (*n* = 33) because of the small group sizes (*i.e.*, type II error). However, it is difficult to propose a convincing mechanism for an association found in women whose only iron deficit appears to be low iron stores because, unlike Hb or sTfR, ferritin is not considered to have a functional role beyond iron storage. The soluble transferrin receptor concentration was slightly, but significantly, higher in women categorized as having low iron stores than in women who were iron sufficient, but adding sTfR concentration to the model did not explain the association. It is possible that the association between having low iron stores and more negative mood was a chance finding resulting from the large number of statistical analyses that were conducted (*i.e.*, type I error). 

Very few studies have examined associations between iron status and depressive symptoms in apparently healthy adult male participants [12,18,24] and none have investigated mood. Two [12,24] of these studies reported that lower iron status may be associated with higher depressive symptoms in men, however, Yi *et al.* [24] only showed a relationship when a non-standard cutoff was used for the CES-D (*i.e.*, >19 rather than ≥16), and the study of elderly men and women by Stewart *et al.* [12] included participants with diagnosed conditions that are likely to have influenced both iron status and risk of depression (e.g., cancer). In contrast, the present study suggests that young men with higher iron status may be at increased risk of higher depressive symptoms, although not at increased risk of depression (defined as a CES-D score ≥ 16). This finding is surprising for two reasons. First, the men in this study had iron levels within the normal range (97.5% were iron sufficient and exclusion of the eight men with serum ferritin concentrations ≥300 µg/L [44] did not alter the results) and very few men reported consuming iron supplements (2.2%) which may contribute to higher body iron levels. Second, the association was not found in young adult women. However, this may be because the distributions of serum ferritin and body iron were considerably lower for women than men. Higher intakes of alcohol are associated with both higher biochemical iron indices [45] and higher depression [46], but it is unlikely that the association seen here is because of a confounding effect of alcohol intake in this student population. Data on alcohol intake were collected daily, and controlled for in the analysis. Moreover, body iron remained a significant predictor of depressive symptoms following inclusion of continuous (alcohol consumption (g/day)) and categorical (≥15 standard drinks/week) interaction terms to account for alcohol intake. Only one other study has reported an association between higher iron levels and higher depressive symptoms in men [18]. However, having analyzed several different iron markers, that study only showed an association with serum iron (an unreliable index to use as a sole indicator of iron status [1]). Furthermore, the association was no longer significant after applying a Bonferroni correction to account for multiple comparisons [18]. It may appear curious in the current study that men higher in body iron showed higher depressive symptoms and stress but not any more negative mood. However, depression is a complex construct and comprises more than just mood symptoms. Emerging research suggests that men have been found to differ from women in their expression of depressive symptoms and are more likely to report symptoms such as aggression and risk-taking [47].

Previous research has documented the detrimental effects of iron overload conditions on physical health, in general [48], and high iron levels in certain regions of the brain have been implicated in neurodegenerative conditions such as Parkinson’s disease and Alzheimer’s disease [49]. This is the first study to find that high iron levels (within the normal range) may be associated with greater depressive symptoms in young men. One explanation for this association may be the generation of reactive oxygen species (ROS). *In vivo* studies have demonstrated that iron generates ROS via the Fenton reaction leading to oxidative stress [50]. Several studies have found that in men and women, higher serum ferritin concentrations were associated with increased oxidative DNA damage [51,52,53]. Furthermore, oxidative stress may contribute to depressive symptoms [54,55]. While the magnitude of the association in the current study was small (a maximum difference between body iron quintiles of 4.2 CES-D points out of 60), the finding that higher body iron was associated with higher depressive symptoms may have important implications for those with even higher iron levels, particularly older men and those with iron overload conditions, and possibly those who regularly consume iron supplements, if a causal relationship is confirmed in randomized controlled trials. 

The current study has a number of limitations and strengths. First, the depressive symptoms measure used in this study, the CES-D, is designed as a screening tool and is therefore not diagnostic of depression per se [30], However, it was not feasible to use the gold standard for diagnosing depression (a structured clinical interview [56]) in the context of this large research study. Second, we did not measure other nutrients that could affect both iron status and mood (e.g., zinc [1,57]). Third, because of the cross-sectional design of this study, causal inferences cannot be made. Fourth, the small number of participants with iron deficiency anemia and non-anemic iron deficiency limited the conclusions that could be made about these groups. The study also has a number of strengths. We excluded participants with evidence of inflammation (CRP ≥ 10 mg/L, AGP > 1 g/L) and controlled for differences in inflammation below the cutoff (using CRP and AGP as continuous covariates). We also controlled for BMI and alcohol intake, health indices particularly relevant to the young adult population and associated with iron status. Moreover, our sample was similar to young adults in other studies in terms of BMI [14] and alcohol intake [58]. 

## 5. Conclusions

This large study, with comprehensive measures of iron status, depressive symptoms, and mood, suggests that low iron status is unlikely to be associated with significant deficits in mood or more depressive symptoms in healthy young adult women. However, this is the first study to suggest that higher body iron may be associated with more depressive symptoms in young adult men. These findings would need to be replicated in another large cross-sectional study, or in a case-control study, before a double blind placebo-controlled randomized trial is conducted to confirm that there is a causal association between high iron status, within the normal range, and depressive symptoms.
